# The European Market for Animal-Friendly Products in a Societal Context

**DOI:** 10.3390/ani3030808

**Published:** 2013-08-14

**Authors:** Paul T. M. Ingenbleek, David Harvey, Vlatko Ilieski, Victor M. Immink, Kees de Roest, Otto Schmid

**Affiliations:** 1 Marketing and Consumer Behaviour Group, Wageningen University, Hollandseweg 1, 6706 KN, Wageningen, The Netherlands; 2 Department of Agricultural Economics and Food Marketing, The University of Newcastle upon Tyne, Tyne, NE1 7RU, UK; E-Mail: David.Harvey@ncl.ac.uk; 3 Faculty of Veterinary Medicine, University St. Cyril and Methodius, Lazar Pop-Trajkov 5-7, Skopje, Macedonia; E-Mail: Vilieski@fvm.ukim.edu.mk; 4 Agricultural Economics Research Institute, Wageningen University and Research Centre, Hollandseweg 1, 6706 KN Wageningen, The Netherlands; E-Mail: Victor.Immink@wur.nl; 5 Department of Economics, Research Centre for Animal Production, Corso Garibaldi, 42-Reggio Emilia, Italy; E-Mail: K.de.Roest@crpa.it; 6 Socio-Economics, Research Institute of Organic Agriculture (FiBL), Ackerstrasse CH-5070, Frick, Switzerland; E-Mail: otto.schmid@fibl.org

**Keywords:** animal welfare, market, European Union, society, state, civil society

## Abstract

**Simple Summary:**

This article takes a future focus on the direction in which social forces develop the market for animal-friendly products in Europe. Although many stakeholders believe that the market is the most viable direction to improve farm animal welfare, economic productivity of the chain remains an issue that on a fundamental level conflicts with the objective to improve animal welfare. The European market for animal-friendly products is still largely fragmented and the differences between European countries are considerable. A more animal-friendly future that is achieved through the market will therefore need substantial policy attention from stakeholders in society.

**Abstract:**

This article takes a future focus on the direction in which social forces develop the market for animal-friendly products in Europe. On the basis of qualitative data gathered in the context of the European EconWelfare project, the differences across eight European countries are studied. The findings suggest that, given international trade barriers that prevent an improvement of animal welfare through legislation, many stakeholders believe that the market is the most viable direction to improve farm animal welfare. Economic productivity of the chain remains, however, an issue that on a fundamental level conflicts with the objective to improve animal welfare. With the help of a deeper conceptual understanding of willingness to pay for animal welfare, the paper finds that the European market for animal-friendly products is still largely fragmented and that the differences between European countries are considerable. A more animal-friendly future that is achieved through the market will therefore need substantial policy attention from stakeholders in society.

## 1. Introduction

Since the Second World War, major changes have taken place in animal production. Animal production has become increasingly industrialized with intensification of production, introduction of new technologies to farmers, high specialization of farms and significant increases in production and the number of animals per farm (*cf.* [1,2]). During more recent years, the society’s awareness of animal welfare and farming issues has grown and been affected by factors such as the activity of animal interest groups, as well as by media attention to animal health crises such as swine fever, BSE, and foot-and-mouth disease. Improvements in farm animal welfare are ultimately grounded in society, and as a consequence the literature on animal welfare has paid substantial attention to the views of citizens on animal welfare. Studies conducted in Belgium [3], Denmark, Sweden [4], Germany [5,6] and the United Kingdom [7,8,9] offer ample evidence of public concerns about animal welfare in Europe. Also Eurobarometer results of 2007 [10] tell us that European citizens worry about the quality of life of intensively farmed animals.

To date, research has not only responded by studying the welfare of animals themselves, but also by increasing our understanding of how different forces in society influence animal welfare. Frameworks have been developed to better understand the influences of, among others, government policies (e.g., [11,12]), production chains [13], markets (e.g., [14,15]), and platforms of stakeholders from these different domains (e.g., [16]). The literature also includes scenarios to understand the complex interactions of these forces in society [17]. The market emerges in most of these contributions as an important channel for the future of farm animal welfare, with most stakeholders agreeing that, within the European context, markets play an important role in further improving farm animal welfare [18]. In a recent contribution to the debate, Ingenbleek *et al.* [19] extrapolate the policy instruments to improve farm animal welfare in Europe, including the instruments that stimulate the creation of a market for animal-friendly products. This work helps policy-makers to identify the instrument that is appropriate in a given condition, but it does not provide insight into whether and how social forces in Europe will create a market for animal-friendly products in the future. 

In line with the theme of this special issue, the present article takes a future focus and offers a narrative on the direction in which social forces develop the market for animal-friendly products in Europe. Drawing on research from the EU EconWelfare project, it first offers a brief discussion about society’s influence on animal welfare. This is followed by findings from an extensive series of stakeholder and expert workshops, which indicate that the market raises both the highest expectations and the greatest levels of concern about the future of farm animal welfare in Europe. As the concerns ultimately seem to pertain to the fundamental contradiction between economic interests and animal interests, the article presents a conceptual understanding of this contradiction, making clear that market development for animal welfare depends on the harvesting of the willingness to pay (WTP) that is created by society. This understanding raises the question whether the development of a differentiated assortment of animal-friendly products in Europe is feasible and currently happening. The article addresses this question by describing the market development in eight European countries. The article discusses the commonalities between the countries and offers conclusions and implications for the further development of a market for animal-friendly products.

In the following, we first discuss the materials and methods employed in this study. Next we present first the societal model as a basis to briefly discuss the workshop findings, followed by the conceptual understanding of the market for animal welfare. Next, we discuss the insights from the eight countries, followed by a discussion and conclusion.

## 2. Materials and Methods

The data for this study were collected within the context of the EU project ‘EconWelfare: Socio-economic aspects of farm animal welfare', which aims to develop policy instruments to support implementation of the Community Action Plan (CAP) on the Protection and Welfare of Animals 2006–2010. The overall objective of the project is to reveal what policy instruments might be effective in the route towards improved animal welfare, to represent the concerns of civil society and to guarantee competitiveness of the livestock industry. In the project, eight (candidate) EU member states are included in the analysis, *i.e.*, Germany, Italy, Macedonia, The Netherlands, Poland, Spain, Sweden, and the United Kingdom. These countries represent different geographical regions of Europe and also represent a rich variety of societies with differences in markets, states, and civil societies. The project consists of several work packages and three “columns” on the animal, the chain and society, respectively, that cut through the different work packages. This article reports insights from the society column. As such it draws on insights and evidence from several research activities of which the most important ones are described below. For more details on the methods we refer to the project reports [20,21,22,23,24].

To answer the question regarding the direction in which the influence of society is developing in the EU, two types of consultation exercises were carried out concerning the subject of policies and issues. The first type of consultation involved a workshop which amongst a diverse group of 36 participants included EU stakeholders from the different participating countries and with different national and professional interests, including academic representatives from the social, economic and animal-based sciences, national policy-makers, animal interest groups, industry, retailing, and farmer organizations. Participants were selected because they all had actively contributed to the animal welfare debate in society before. At the workshop, participants were asked to focus on the external circumstances that would constrain or favor animal welfare initiatives in Europe. They each wrote down five constraints on red post-it papers and five positive developments on green post-it papers. Next, because conditions may vary within Europe, participants were asked to paste the papers in the European region to which they pertained. They could choose between Sweden, UK/The Netherlands/Germany, Poland/Macedonia, Spain/Italy, and the EU in general. Two researchers from the Econwelfare project grouped similar statements together and then led a discussion in which they invited the participants to explain or elaborate on the constraining and positive developments that they had written down. Notes were taken from the discussion and the papers with the post-it notes were taken for further analysis.

The second type of consultation was the organization of a European seminar of major retailers and animal interest groups from different EU countries. The retailers were invited because they represent the demand of consumers, and the animal interest groups were invited to represent the part of society, which has the highest sensibility towards animal welfare. The retailers were represented by participants of large retail businesses (Royal Ahold, Coop Italia, Coop Sweden/Svenskhandel, and two participants of Carrefour, France), one national representation organization of food retailers from The Netherlands (Centraal Bureau Levensmiddelenhandel), two European umbrella organizations for retailers (Eurocommerce) and the meat industry (European Liaison Committee for the Agri and Agri-Food Trade), respectively, as well as one standard-setting organization for retailers’ procurement criteria (Global Gap). Animal interest groups included representatives of the German and the Dutch societies for the protection of animals (the Dierenbescherming and the Tierschutzbund, respectively), the German organization Provieh, Compassion in World Farming and the Eurogroup for Animals. The latter is a European representative for the major national animal interest groups in Europe. The groups first had separate discussions, which were followed by a plenary session that clarified the convergences and differences of opinion. The discussions were structured around four statements that were developed after reviewing literature on the role of farm animal welfare in society. The statements were: (1) Higher levels of animal welfare should be achieved primarily through mandatory EU regulations. (2) Farmers and farmer groups will only work towards higher animal welfare if there are sufficient financial incentives. (3) Voluntary animal welfare schemes combined with labeling are the most effective in raising animal welfare as they act through the market mechanism. (4) The best way to change consumers’ buying behavior is to educate and inform them about animal welfare. All discussions were led by researchers of the Econwelfare project. Other researchers took notes and photographed important output materials, like flip-over pages. These notes and materials functioned as input to a report [20] and the present article.

The question of how the market for animal-friendly products is being developed, is answered on the basis of input from the different countries. For each country, extensive country reports were written that summarized the available country-specific information derived from desk research, expert interviews, and stakeholder workshops [21]. Topics covered included (*inter alia*) the opinions of animal scientists, citizens, and actors in the animal production chains on animal welfare initiatives. Furthermore, the authors held in-depth interviews with the researchers that had conducted the research to get deeper insights into the background and more details of the provided information, and to minimize the chance of interpretation biases. All these interviews were recorded and transcribed (see [22]). In addition, researchers selected the most important initiatives that (directly or indirectly) aimed at improving animal welfare based on desk research, expert interviews and prior knowledge. This yielded a total of 84 descriptions from a rich variety of societal contexts (for a more elaborate description of these data and more results see [23,24].

In addition to these methods, the results of which will be described in some detail in this article, a number of other investigations in the EconWelfare project also inform the discussion of the societal influences on animal welfare. These include, among other things, inductive investigations on policy instruments [19,25], a Delphi survey of stakeholders [18], a belief network analysis of stakeholders [26], and a costing survey [27].

## 3. Society’s Influences on Animal Welfare

**Figure 1:**
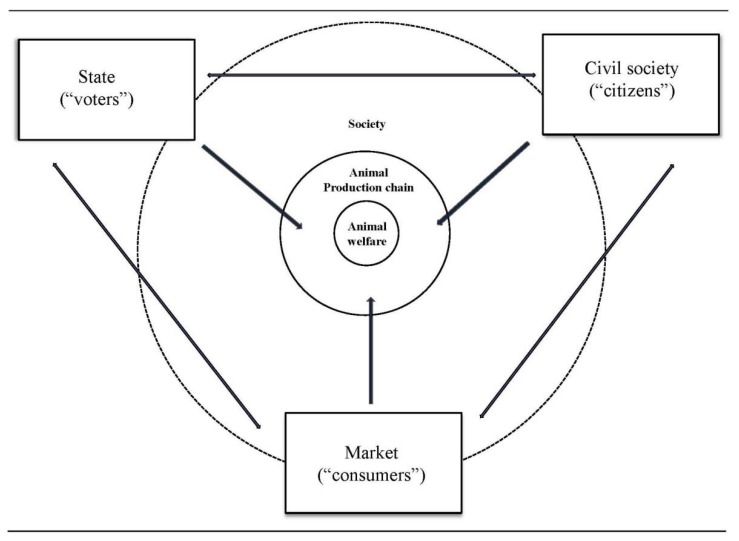
Society’s influences on animal welfare (based on [31]).

Widely used definitions of animal welfare state that welfare is about an animal’s ability to cope with its environment [28,29]. When applied to sentient animals, animal welfare is therefore about how animals feel, see also Avéros *et al.* [30]. This sets the animal in the center of any study on animal welfare, including those that focus on the business chain and society at large. The definitions also suggest that animal welfare is in the first place influenced by its *direct* environment. For farm animals, this environment is largely determined by the production chain (*cf.* [13]), including all chain members that directly influence how the animal feels—the primary animal caretakers: farmers; transporters; and slaughterers—but excluding members that have no direct influence on how the animal feels—meat or milk processors, retailers, and consumers. The primary animal caretakers create the direct environment for the animal, which influences the abilities of animals to cope with that environment. Everything else has an *indirect* effect on animal welfare at best, and for the sake of simplicity we refer to the sum of these factors that influence animal welfare indirectly as *society*. In short, societal influences do not have a direct impact on animal welfare, but they influence the behaviors of actors in the animal-production chains, which in turn influence animal welfare (see also the conceptual framework in Figure 1).

We draw on a societal model that divides the concept of society into three institutional domains: state, market, and civil society (see also [31]). Each of the domains employs its own specific logic, rationality and ideology, and has organized itself on the basis of different ‘rules of the game’ or ‘institutions’, with its own coordination mechanism. Government shapes society by coordination and regulation and as such it also influences the direct environment of the animals through setting and organizing control for regulations. 

The market sector converts inputs into outputs led by competition within the legal boundaries. By making animal welfare part of the competitive process, it can make investments to improve the conditions of animals above legal standards. Strictly speaking, animal-based production chains that shape the direct environment for animals belong to the market component, because the environment that determines animal welfare is created by businesses. However, for the purpose of this article, we separate the chain into two parts and refer to the market as only those actors that belong to the indirect environment of the animal (downstream of the slaughterhouse or packing station), which includes any organization, group, or individual that does not deal with living farm animals directly. 

Civil society includes social relations among citizens, either in organized networks like animal interest organizations, or informal networks of family and friends, facilitated or not by (social) media. Through such social pressure, people that work with animals may take better care of them. The next section will discuss which of these directions according to experts is most important for the future of animal welfare in Europe.

## 4. How does Society Direct Farm Animal Welfare in the EU?

The experts that participated in the EU workshops in particular brought up issues that pertained to the state and market influences on animal welfare, while the role of civil society was more addressed in terms of indirect influences exercised through state and market. We discuss these below.

### 4.1. Moving towards a Clearer Legal Basis for Animal Welfare

Several experts that participated in the EconWelfare workshops indicated that improvements in the measurement of animal welfare provided an opportunity to improve animal welfare. The current legislation is, according to some experts, too detailed but, according to others, contains significant gaps. The concepts, definitions and terminology are, however, becoming clearer. Several experts in the workshops also saw positive developments in the infrastructure that is needed to improve animal welfare: less bureaucratic standards; increased integration of animal welfare with food quality standards; more efficiently integration throughout the food chain; improvement of training for farmers and transporters (although one noted that the training still differed widely within the EU). Among retailers and animal interest organizations, a consensus emerged about the fact that the enforcement of existing legislation is necessary in order to increase animal welfare to the minimum legal level. According to the retailers, a public-private partnership should be established to streamline the systems of control of legislation, entrusting the control to third parties [21]. This may help to prevent confusion of the roles: farm advisors are, for example, sometimes also farm inspectors.

These opinions suggest that there is still a debate on what the legal level of animal welfare should be in the EU, but the debate seems to be decreasingly hindered by confusion in concepts, terminology, and measurement. Such efforts will ultimately help to release the potential for animal welfare improvement in Europe, because they establish a clear and common basis for animal welfare throughout the EU from which further improvements can be developed. As discussed below, these improvements should not only come from the direction of state, but perhaps even more so from the market, and thus from civil society.

### 4.2. A Move towards the Market: Stakeholders’ Views on Developing Animal-Friendly Consumers

Overall, there seems to be a growing consensus among the experts that although a clear and common legal basis is important, further improvements in animal welfare are expected from the market domain. Important reasons behind these expectations are the WTO regulations and international trade restrictions that constrain governments in their ability to protect their markets from cheaper, less animal-friendly, import products. Meanwhile, economic growth and higher living standards will increase WTP of consumers for animal welfare, given that sufficient public awareness exists for the issue, thus creating a market opportunity to improve farm animal welfare. All stakeholders expressed that public awareness was increasing, due to, amongst others, animal-interest group campaigns and media attention. Commercial parties, especially retailers, have responded in their Corporate Social Responsibility (CSR) policies and are now competing on the basis of animal welfare, thereby further bringing animal welfare to the attention of the consumer. Even in countries that traditionally depend on the state to make improvements in animal welfare (such as new and candidate EU member states), a young and urban middle class is rising that appears to have some WTP for animal-friendly products. 

With the rising awareness new problems are also appearing: animal welfare as an issue is increasingly competing with other issues. Animal welfare is, according to experts, however, suitable to connect to other social concerns, such as environmental issues (as for example practiced in organic farming), and is also connected to quality (where improved animal welfare strengthens higher quality perceptions of consumers). In order to pursue such a strategy, animal interest groups may, however, have to compromise with other interests because animal welfare can in some cases conflict with food safety and sustainability requirements (for example, ‘wasting’ natural resources on animals, and ruminant animal contribution to methane release as a greenhouse gas).

While agreement seems to exist that the market is of paramount importance for the future of farm animal welfare in Europe, many experts also point at the potential conflicts between animal interests and economic interests, in particular with respect to the question how the costs for animal welfare are covered. According to an expert as well as to several participants in the workshops, farmers experience cost increases due to animal welfare that are not covered by higher prices. While the sharing of costs and benefits of animal-friendly products within the chain is an issue that remains on the table, most comments seem to refer to the question: How can we get consumers to pay more for animal welfare? Both animal interest groups and retailers therefore strongly stress that education and information on animal welfare are crucial for consumers. They believe that consumers are still not informed enough: They are driven by emotions and have idealistic pictures in their minds with regard to animal welfare. Labeling is not the only or easiest solution to this problem because labeling is frequently unclear to consumers and animal welfare is too complex an issue to fully inform consumers. One solution offered is that public authorities need to promote initiatives involving especially children and young people.

In summary, the stakeholders see the market to be of paramount importance for the future of farm animal welfare. At the same time they recognize barriers of which the potential conflict with economic interests is the most important one. To solve this conflict, a solid conceptual understanding is indispensable. We turn to this conceptual understanding in the next section. 

## 5. Conceptual Understanding of the Conflict between Animal Welfare and Economic Interests

**Figure 2:**
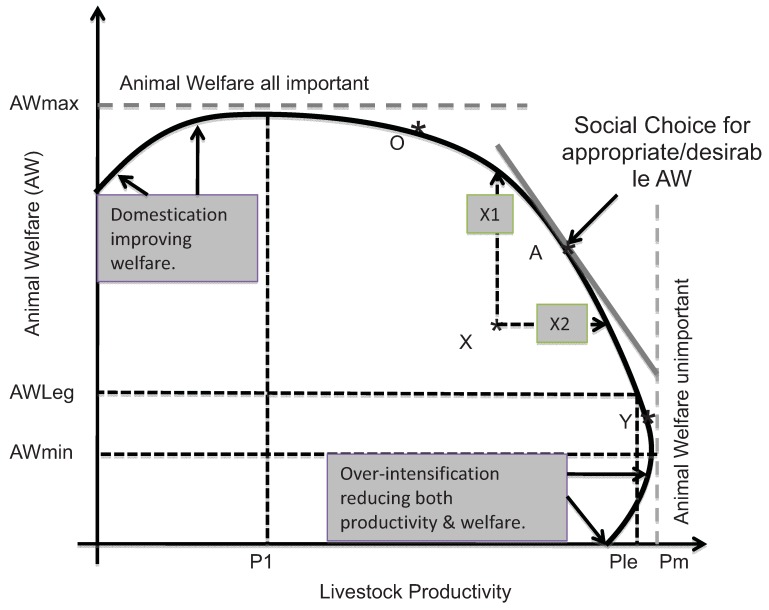
A possibility frontier for animal productivity and welfare (based on [32]).

With respect to animal welfare, the conflict with economic interests often occurs because the objective of maximizing profits from farm animal production is, perhaps, fundamentally in conflict with the objective of maximizing animal welfare. Hence, we focus here on the relationship between productivity and animal welfare (Figure 2) following [32], and as also used by [26,33]. This diagram illustrates a conceptual model of the behavior of the animal products supply chain, from an economical perspective. Firms and farms in the supply chain can be viewed as competing with each other to provide the products demanded by consumers, subject to the social and legal constraints imposed by citizens through their governments and by the prevailing social norms. As these firms strive to make and improve their livings (profits), they necessarily exploit animals. Typically, the more intensively animals are farmed, and the more insensitive the slaughtering processes, the worse is animal welfare—battery hens and intensive pig rearing systems are the most obvious examples. On the other hand, unless animals are treated reasonably well, they will not thrive or produce quality carcasses, so animal welfare is not always and necessarily inconsistent with tolerable levels of animal welfare. The competition between firms in the supply chain can be thought of as generating a ‘best practice’ frontier (termed a production possibility frontier), which relates livestock productivity to associated levels of animal welfare, as shown in Figure 2. Along this best practice frontier, greater livestock productivity is generally associated with lower animal welfare. The model is clearly conceptual rather than practical, since neither animal welfare nor livestock productivity is presently reducible to unambiguous single measures. 

Nevertheless, the implication of this conceptual model is that competitive supply chains generally find that improvements in animal welfare are associated with reduced animal productivity (and associated increased production costs). Of course, not all firms in the supply chain perform up to industry best practice—so some, perhaps many, will not be operating on this frontier of best practice at any given time, but will lie somewhere within it (at a point like X). These less efficient and effective firms could improve both animal productivity and animal welfare at the same time (moving to Segment A, between X1 and X2 on the production possibility frontier). In principle, the continual processes of supply chain competition are expected to encourage these currently inefficient firms to move closer to the PPF for animal welfare. Without this continual competition for market shares and margins over costs, the pursuit of improved animal welfare is likely to be slow. In addition to competition within the supply chain, the more R&D and effective information, dissemination and extension services are available, the more rapidly such best practices can be expected to percolate through the industry, and the more rapidly can the best practice frontier be expanded (shifted upwards and to the right in Figure 2).

Majewski *et al.* [27] document in detail the cost consequences at the farm level of a range of stylized but detailed higher animal welfare (AW) standards, as collections of specific norms relating to animal conditions and management practices. For all the standards examined, with the exception of dairy cows, the higher standards generated increased net costs (net of the benefits achieved by adopting some of the norms). In the dairy sector, at least in some countries, the research conducted by the EconWelfare team suggests that the typical dairy farm is actually currently X-inefficient in terms of Figure 2, and able to improve animal welfare and reduce production costs (improve productivity) simultaneously. Given the recent competitive pressure on dairy farms in the EU, it is to be expected that such improvements will happen as the sector adjusts towards more efficient operations [27].

First, assuming that they are properly enforced, rules and legislation set the minimum levels of animal welfare, at AWleg (Figure 2). AWleg may also be interpreted as the level of sector-wide standards that aim to secure the legitimacy of the entire sector, if these happen to be higher than the actual legal standards. Without new technologies and techniques to improve animal welfare and productivity at the same time, introduction and enforcement of a new higher level standard for AWleg—shifting this restriction to the left—will force production and supply chain practices up the best practice frontier, implying reduced economic productivity with higher costs of production. 

Increased enforced standards at AWleg will, however, encourage adaptation and innovations to improve productivity given this new minimum standard, so that over time and other things being equal, the system will tend towards Ple/AWleg in Figure 2. These adaptations and innovations will generally respect the minimum level of animal welfare standards which society is prepared to tolerate. As a consequence, the frontier of welfare/productivity combinations will tend to adjust to this minimum standard, as long as consumer demand confirms that society is prepared to enforce the minimum standard and, as a result, pay any necessary premiums to enable the chain to comply effectively. If not, then the sector will contract in the face of non-compliant competing supplies until the remaining firms and businesses can afford to comply and stay in business—the domestic market frontier will shrink to meet the effective market at the enforced minimum standard.

Second, society signals its preferences through its WTP for animal welfare. At a macro level this is expressed through acceptance of higher AWleg standards as well as through sales of differentiated animal-friendly products. The latter are important because a society may consist of different groups that have different levels of WTP. Offering different products associated with different levels of animal welfare may help to unlock the total WTP for animal welfare in a society. As societies become richer, better educated and more able and willing to take care of their environments and activities, so they tend to be more WTP for improved animal welfare (as outlined in [32] and illustrated in [25]). In the terms of Figure 2, the signals are transmitted as the ‘price’ of animal welfare relative to the price of animal products (*i.e.*, the premium paid for differentiated animal-friendly products). The rising share of free-range eggs (and chicken) at the expense of cage eggs is an obvious and outstanding example. In societies where animal welfare is of no importance at all, animal production systems tend to be driven towards maximum productivity and minimum animal welfare (for any given species and for the sector as a whole, at AWleg)—characterized by [26] as the vicious circle of animal welfare degradation. At the other extreme, a society, which considers animal welfare to be all-important would tend to drive its production system towards the maximum level of animal welfare (and consequently a relative minimum level of animal productivity—with associated higher relative prices for animal products).

If governments (or standard organizations consisting of actors from the market and civil society) decide to try and impose higher minimum standards than their society is willing to pay for—pushing AWleg higher up the frontier than the (implicit) socially preferred point (A), then some consumers may respond by seeking to source their animal products at lower cost elsewhere, or simply reducing their consumption. The end result may be that the attempt to impose higher standards could drive domestic producers out of business in favor of imported products (with possibly lower animal welfare standards). In a closed society/economy, where no such alternative sources of production are available, the imposition of such ‘forced higher standards’ would result in higher costs (and prices) for animal products relative to other goods and services, which would tend to reduce consumption and production, and thus reduce the size of the sector (and hence shrink the frontier). The combination of higher price and lower consumption demonstrably reduces social welfare, other things being equal, relative to the condition prior to the introduction of the enforced higher standard. However, in the event that consumers are willing to pay for these higher standards, then the higher prices will not lead to a decline in consumption. This is the standard outcome of interference and intervention in a competitive market, which otherwise delivers socially optimal outcomes.

Of course, as Harvey and Hubbard [33] demonstrate, WTP for improved animal welfare is problematic, especially as measured with partial and focused choice experiments. In practice, consumers’ signals about what they are willing to pay for improved animal welfare are confused by their other activities and priorities. People do not always make the time to consider animal welfare specifically when making their purchase decisions, and may be more likely to bundle animal welfare considerations with other attributes of animal products, such as quality and provenance. Indeed, some consumers may not wish to be reminded of animal welfare considerations at all when purchasing animal products. However, a competitive supply chain can be expected to continually test consumers’ responses to different offers of animal products, including their welfare provisions, so as to exploit the latent WTP as fully as possible. Otherwise, the supply chain firms risk losing market share to other more competent firms. Even those people who regard all animal welfare as sufficiently important to avoid consuming animal products altogether exert their market influence by reducing the demand for these products. To the extent that these views become more widespread, the demand for animal products will decline until or unless the supply chain responds by improving animal welfare.

In short, this conception of the operation of a competitive market indicates that improved animal welfare will tend to reflect increased consumer awareness of animal welfare issues and from the abilities and capacity of the supply chain to respond to these pressures and deliver the higher premia available from the supply of better animal welfare. Differentiation of products associated with differing levels of animal welfare is a critical element of these market processes.

## 6. The European Market for Animal-Friendly Products

This understanding of how the exploitation of WTP works in a market raises the question whether the development of a differentiated assortment of animal-friendly products in Europe is feasible and currently happening. This section explores the current differences and recent trajectories in European markets in order to understand how these develop a WTP for animal welfare.

### 6.1. UK: Europe’s Template for Developing a Market for Animal-Friendly Products

The UK represents in many ways a template for how a market for animal-friendly products can be developed. The country is characterized not only by a strong presence of a conscious consumer segment, but it also has a high level of general awareness for animal welfare. Animal welfare appears to be an important issue for UK consumers, as 66% answered in a 2001 Welfare Quality^®^ investigation that they had reduced their meat consumption due to their concerns regarding treatment of animals in 2001 [34]. In that respect, it can rely on a civic sector characterized by several professional animal-interest groups of which the Royal Society for the Prevention of Cruelty to Animals (RSPCA) is best known. Celebrity chefs have also increased the animal welfare concerns among the public. While the animal-interest groups in general take a critical stand towards the chain, they are also represented in animal welfare forums in which industry members and government representatives also appear [35]. 

WTP for animal welfare is harvested by schemes for animal welfare, in particular Freedom Food, which positions itself above legal, but lower than organic standards [20]. Freedom Food is available in several supermarkets and has increased its assortment to over 900 different lines of Freedom Food labeled products [36]. Next to Freedom Food, also several store brands of supermarkets have adopted above legal standards for several animal-based products [34]. As such, there is an upward pressure in the chain to further improve animal welfare that stems from competition between retailers and brands that aim to differentiate themselves on the basis of animal welfare and other issues for which standards are developed. This process of competition helps in the respect of “harvesting” the WTP potential that is present.

In short, the UK has developed the most mature market for animal-friendly products. The question for the future seems to be: Where do we move from here? According to one expert that we interviewed, there is a level of fatigue rising with regard to the animal welfare issue. One way to deal with this is to make animal welfare part of a broader set of standards that assure legitimacy on a number of different issues, including for example environmental degradation and unfair international trade relationships, “Buy British” and, in particular, food safety [34]. 

In the UK, the chain as a whole has little history of collaboration and chain relationships are characterized by mistrust. The low level of trust and collaboration is also shared by the government and animal interest groups. According to an expert, the animal welfare forums are described by some participants as attempts of the government to keep an eye on the industry, rather than as a source of innovation.

### 6.2. Sweden: From Legislation to Market

Citizens have high levels of trust in Swedish authorities concerning inspection and control of production of meat products, not surprisingly considering the Swedes perception and expectations of government authorities in general [37]. Before entering the EU, Sweden therefore ensured relatively high levels of farm animal welfare through legislation. After entry into the European Union, politicians in Sweden agreed to maintain the legal standards [38]. Some Swedish rules are more stringent than EC directives whereas others concern specific topics not ruled in EC directives and match many of the most important aspects [39]. However, these higher standards potentially make the domestic sector vulnerable for imports of cheaper products produced at lower legal standards.

The challenge for Sweden for the future seems to be to turn the importance that Swedish consumers attach to animal welfare into WTP. In other words: They need to strengthen their market proposition of the Swedish products over the cheaper imports. Swedish consumers need to become aware that they cannot rely on their government to accommodate all their concerns, but that they also should “vote” as consumers. The Swedish origin is then an important marketing instrument. In Sweden, at least 70% of the consumers say it is very important that food is produced in Sweden, in particular because of AW concerns [34]. Animal welfare can therefore be combined with region- or country-of-origin labeling, and environmentalism. A label like Swedish Seal of Quality can therefore add value to Swedish products. A growing number of people also take responsibility themselves and choose products on the basis of attitudes and values, also taking production conditions of animals into considerations [34]. The latest poll has shown that 52% of Swedish consumers are aware of the label. Relatively few Swedish farmers are, however, connected to the Swedish Seal of Quality [40]. Some other initiatives resonate more strongly with farmers. The Swedish Broiler Welfare Program that allows poultry farmers to apply stricter stocking density limits has been successful because 99% of the broiler producers are part of the initiative [35]. 

Sweden also has a relatively well-developed structure of animal-friendly organizations, thus enabling the development of new animal-friendly products and labels. However, some organizations take a critical stand towards businesses including those that introduce animal-friendly products to tap the available WTP. The label “All is well” was, for example, seriously damaged by recent television documentaries that showed animal welfare problems in farms that complied with the legal standards. Such media attention undermines the efforts of the Swedish quality label.

### 6.3. The Netherlands: Exporting the Polder Model?

The Dutch have followed the template set by UK in several ways. Animal interest groups in the country have professionalized and while some of them have specialized in putting pressure on the chain, the largest organization (the Dutch Society for the Protection of Animals, hereafter DSPA) has become a strong collaborator with industry and retailers. Animal welfare has become part of the competitive process between brands and retailers. The DSPA has stimulated this process by developing a labeling system, in which products are graded with stars depending on their level of animal welfare (usually with three stars for organic products and one star for products that are slightly above legal levels).

The Dutch, however also differ from the template set in the UK. First, The Netherlands are among the largest exporters of livestock products in the world (in particular pork, poultry and dairy). This puts constraints on the development of the middle market for animal welfare, because the production for their domestic market may lose important scale effects if it is set apart from the production for export markets at lower standards. It also poses the chain with an important legitimacy risk, as consumers may wonder why they pay more for their animal-friendly products while the same companies continue to produce animal unfriendly bulk for export markets.

Second, the Dutch have a strong tradition of collaboration, both within the chain and between the for-profit and not-for-profit sectors, which they call the “polder model”. The DSPA is, for example, heavily involved in innovation projects in which animal-friendly husbandry systems are being developed. Such projects are in several cases supported by the Dutch government, which pays, for example, for technical and market research at the early stages of the project. Although some of the projects fail, the different parties are likely to team up again in future projects, which develop their collaboration based on previous levels of trust and communication skills. As compared to the UK, the Dutch sector is therefore relatively vibrant and innovative in developing the midrange market segment.

The key question that Dutch are confronted with for the future of animal welfare is how they will align the interests of the export market with the innovativeness on the domestic market. While the differentiation and harvesting of the available WTP on the domestic market has clearly started, the export sector still has a strong focus on cost price and bulk. While the export sector has currently only been able to respond to strong and clearly articulated demands for animal-friendly products (such as for animal-friendly bacon from the UK), it will still need to learn to develop animal welfare more proactively. In order to also harvest the potential for WTP for animal welfare in its export markets, they need to find ways to export not only their livestock products but also their polder model.

### 6.4. Germany: the Future Breakthrough?

With its central position in Europe and high level of purchasing power, the German market probably fulfills a key position for the future of the European market for animal-friendly products. The country has a large urban middle class that has some concern for animal welfare. Government legislation is above the minimum requirement of the EC, and the directives cover many welfare criteria and match some of the most important aspects [39]. The country has both animal interest groups with a critical position towards the chain and some that are open for collaboration. The groups are, however, more focused on legislation than on the market [36]. German citizens are broadly concerned about animal welfare; the protection of animals has recently even been integrated into the German constitution. Germany will continue to adopt new AW regulations, even if the EU does not have specific regulations for that topic [21].

While concerns may be reflected in the legislation, they are not (yet) reflected in the market. The current level of animal welfare differentiated products is however relatively low. Competition between brands and retailers in the mainstream focuses on price and product quality rather than animal welfare. Next to organic products, several initiatives exist, such as Neuland, that offer animal-friendly products to small retailers with short supply chains. This offers some differentiation to the retailers that aim to avoid price competition with the large supermarkets. Some of the supermarket chains have their own animal-friendly labels, but they only have a few requirements that cannot always be traced back easily. Despite the development of alternatives, their total market share is assessed to be below one percent. The initiatives also receive relatively little marketing support in terms of promotional campaigns and the like [39]. There is therefore still a clear gap between organic and mainstream products.

### 6.5. Italy: Where North Meets South

Moving south from Germany and arriving in Italy, the market potential for animal-friendly products is at first sight not so different. Ethical considerations about animals’ conditions of life are accompanied by the suspicion that bad living conditions in livestock farms could result in potential problems for human health [39]. One third (of 60 interviews carried out in 2001) claimed to have reduced their meat consumption because of animal welfare concerns due to meat scares related to BSE. As a consequence, concerns about animal rights and animal welfare issues have grown [34]. The organic market is also relatively well developed, in particular in the cities in the North (although most organic products are fruit and vegetables). In general the motivations for buying organic food are food quality, health and the environment, but not (yet) animal welfare [34]. Retailers have anticipated animal welfare issues in the media and have developed farm standards that they can implement as soon as they consider the time to be ripe.

Nevertheless, Italian consumers are less concerned about animal welfare than consumers in northern Europe [38]. As became clear from studies in the first years of the Welfare Quality project, Italian consumers are not very concerned about animal welfare. Italian citizens mostly refer to animal welfare because of the impact of animal welfare on human health. This is reaffirmed in the consumer request for healthier and tastier products: low animal welfare level could mean low quality of food [34]. 

The animal-interest groups in Italy have a much weaker position compared to the Northern countries. As a consequence, they fail to raise media attention for animal welfare issues and they play a little or perhaps no role in the development of standards. Although retailers have invested in farm animal welfare standards it remains unclear to what extent and how animal welfare is addressed.

### 6.6. Spain: The Southern Model

While in Italy the level of animal welfare is relatively low when compared to Northern Europe, in Spain a general awareness among consumers on animal welfare is largely restricted to a small but gradually increasing group of involved consumers in big cities that is also concerned about ecological problems and food safety. A few regional initiatives, an example is EcoVera that produces organic eggs for the regional market, have been successful in improving welfare and creating awareness [24]. Most farmers and retailers think, however, that implementation rules and standards on animal welfare imply more expenditure in their farms or businesses. Nobody has explained to them the reasons, implications, and benefits to introducing animal welfare measures in their farms or businesses.

Animal-interest groups reflect the low level of concern regarding farm animal welfare. In Spain there are 284 Non-Governmental Organizations related to defense and protection of animals, according to an investigation of the Spanish project partner. Most of them are local organizations, dedicated to care and protect pets, such as cats and dogs.

### 6.7. Poland: Catching up?

Although a small market for organic products has begun to develop among young higher income consumers in Poland, this demand is still low. Likewise, civil society’s influence on animal welfare is still very small. Klub Gaja is the most popular societal group representing animal interests. It has initiated several campaigns (like “Do you know what you eat?”) that were highly successful in creating awareness among citizens. Nevertheless, the influence on production and demand was limited [24]. Most Polish supermarkets do not offer animal-friendly products. As they often do not have a formal Corporate Social Responsibility policy, they do not intend to introduce them into their assortments in the future either. Within civil society, the focus is much more on the rapid socio-economic changes that are taking place, making animal welfare an issue of minor importance.

On the bright side, Poland has developed a professional advisory system for farmers. Farmers have received training on animal welfare in which many farmers participated [24]. These assets are important for the starting development of animal-friendly products or when Polish farmers aim to comply with foreign standards for animal-friendly products on export markets like Germany.

### 6.8. Macedonia: Seeing Opportunities?

In candidate-EU member state Macedonia, upward pressure to improve welfare comes from the necessity to meet the minimum EU legislative requirements. These are, however, not always considered a burden to productivity. In particular in Macedonia the participants of the stakeholder workshops emphasized that improving animal welfare legislation was a means of getting the issue of professionalizing animal production chains on the agenda. In terms of the trade-off between animal welfare and productivity, there is still sufficient space to improve the imperfections in the chain. Hence, the stakeholders hint at solutions in which both productivity and animal welfare can be improved simultaneously. In this respect they expressed a clear need to improve the education of chain members.

## 7. Discussion

The narrative on the market development for animal-friendly products in eight European countries brings up at least four issues that require attention in policies that aim to develop this market further in the future. These issues pertain to the market segments across Europe, the role of the exporting countries, new product development, and the sharing of costs and benefits of animal welfare in the chain. We discuss these subsequently.

### 7.1. Market Segments across Europe

A first important observation resulting from the project is that the European market for animal-friendly products can be roughly divided into three segments (see also De Jonge and van Trijp [41]). First, there is a usually relatively small market segment of conscious consumers that deliberately choose food products that stand out in their social contribution. These products are mostly organic, and include not only above-legal standards of animal welfare, but also pay closer respect to environmental impact and biodiversity. The conscious consumers are more numerous in the wealthy areas of Northwestern Europe, but are also present among the young urban professionals in cities in the New Member States, such as Warshaw and Skopje. The organic regulation that supports the conscious market offerings of their preference is similar across Europe and anchored in the EU legislation [35]. This makes organic in fact a pan-European brand trusted by consumers and facilitating cross-border trade.

Second, in each of the countries a relatively large segment seems to exist that sees animal welfare as an issue that should be solved by retailers and other players in the food supply chain. They usually purchase the mainstream products, but they may get upset when scandals appear that animal welfare regulations are violated in the chain or when those regulations fall short with their expectations. Also this group is relatively homogeneous across Europe, at least in those areas where major international supermarket chains dominate the food market. The unified European legislation for animal welfare has in that respect been incorporated by Global Gap, a standards scheme of purchasing criteria, which is adopted by most European supermarket chains. Although Global Gap does not set any above legal standards, it provides extra control on compliance with the legal standards on top of the checks exercised by governmental bodies [42].

Finally, a group seems to exist that may be willing to pay more for animal welfare, but would not go as far as the conscious consumers in that they are willing to pay for organic products most of the time. This third group shows most variation across Europe. It consists of people that may be willing to deliberately choose animal-friendly products with above legal standards, as well as of consumers that follow their favorite brands and supermarkets in their decision to upgrade the assortment to higher levels of animal welfare. An inventory of initiatives to improve animal welfare found 26 initiatives that aim to develop the market for animal-friendly products between organic and mainstream [24] (p. 32). Almost all initiatives had a national focus, of which some even focused on specific regions within a country. Whereas the initiatives to improve animal welfare within the other two segments therefore have a strongly European, or otherwise international, character, the “middle market” between organic and mainstream is mainly developed by within country initiatives. This potentially hinders cross-border trade, as consumers may not understand the animal welfare claims that imported products make. As the market size of national and regional markets is generally smaller than that of international markets, the chain may not exploit the potential scale advantages associated with larger market size (like a reduction of shared costs for product development, development of criteria and control).

### 7.2. The Role of the Exporting Countries

The fragmentation of the market is problematic for the future improvement of animal welfare in Europe, because the production is concentrated in several exporting countries that produce more than they consume (in particular The Netherlands and Denmark). In an absence of more homogeneous standards to develop the middle market in their exporting countries, producers may refrain from any improvements at all. Adopting a large range of different schemes may be at the expense of scale advantages that are required to keep costs under control (which in turn may be necessary to prevent cost increases beyond the WTP). The Welfare Quality^®^ project has aimed to develop measurement instrument for animal welfare that could in principle be used by all these different schemes and initiatives. Even if the different schemes would set different standards for animal welfare, the underlying dimensions would be comparable. To date, these organizations have, however, not shown much appreciation for the measurement instrument. A recent study by Aramayan and colleagues [43] found that they basically showed interest in adopting different parts of the monitor, rather than taking the whole package. The middle market for animal-friendly products in Europe is therefore unlikely to become more homogeneous across Europe on the basis of the existing initiatives.

### 7.3. New Product Development

Although the future of farm animal welfare depends increasingly on the market, this does not always result in the development of new differentiated animal-friendly products that may help to harvest the available WTP. In some countries the position of the animal-interest groups is not (yet) strong enough to engage in product development (either for export or for the home market), while in other countries the traditional division between market and civil society has not (yet) made place for an atmosphere of collaboration from which a wider assortment of animal-friendly products may flow.

According to the results of the stakeholder workshops, the animal interest groups believe that the retailers are the best actors to push products with a higher level of animal welfare. They also recognize that retailers are increasingly willing to take “ownership” of animal welfare brands because they aim to strengthen consumers’ trust in their brands. At the same time, animal interest groups denounce the lack of transparency in communicating the contents to the consumers. They call for clear animal-based indicators to set standards [25], comparable to what is advocated by the Welfare Quality^®^ project [44]. The retailers in turn believe that the voluntary schemes promoted by the animal interest groups are relevant, but run the risk of being restricted to niche markets and thus not being effective in harvesting the WTP that society creates.

### 7.4. Sharing Costs and Benefits of Animal Welfare in the Chain

An increased WTP for animal welfare can be translated into financial incentives for farmers. That does not mean, however, that it will happen automatically. The costs of standards are a constraint according to several experts. They warn that WTP does not always translate into incentives for farmers, or that incentives are not always in line with the (type of and level of) investment that is required from farmers. One expert notes that it is not always clear who bears the risks of improving animal welfare and another witnesses that in the early stages of initiatives the numbers of farmers who participate only increases slowly. Farmers increasingly get the impression that their animals’ welfare is found to be more important in society than the welfare of the farmer, according to one. Retailers see the need for public subsidies when farmers have to introduce system changes in their farms [21]. However, as Harvey and Hubbard point out [26,34], if public subsidies are warranted by genuine market failure, then it makes more sense to subsidize the consumption of animal welfare friendly products, rather than their production. Not only would consumer subsidies encourage the development of improved animal welfare supply chains, but they would also be more consistent with international trading obligations. In addition, these subsidies would only be paid to the extent that consumption (and thus production) of improved animal welfare products actually increases.

However, farmers are faced with the empirical fact that only few retail chains pay the farmers a higher price for their higher investments in high levels of animal welfare (except for organic farms and some niche standards like Neuland in Germany and Freedom Food in the UK) [23]: In this respect the animal interest groups in the seminar stressed the need for a better distribution of costs and benefits among the actors of the supply chain in order for the consumer’s WTP to reach the farmer who bears the costs of meeting animal welfare constraints. Retailers, but to a certain extent also animal interest groups, share the opinion that there are still many “imperfections” that have potential to raise animal welfare and productivity simultaneously. In principle, market competition should ensure that all actors in the supply chain are sufficiently well rewarded for their efforts that they are willing to remain in the industry and continue to produce and supply. However, it is clear that there is far more market power at the retail (and processing) ends of the chain than there is at the farm level. In this sense, more general competition policy, which polices abuse of excessive market power, is at least as important as specific measures to improve animal welfare.

## 8. Conclusion

In Europe, social forces are developing a market for animal-friendly products. The direction of the market is important because stakeholders see international trade restrictions as an important barrier for substantial improvements in animal welfare via legislation. While in some countries the process of market development has led to the development of an assortment of differentiated animal-friendly products that tap different levels of WTP for animal welfare, in other countries the development is still at more rudimentary stages. Although companies contribute to the development of an assortment of animal-friendly products, products need legitimization and active involvement from animal-interest groups. Not all European countries have a civil society on animal interests that is willing and/or able to contribute to such product development. While the organic community is well-organized in Europe in terms of common standards that facilitate cross-border trade, initiatives to harvest the WTP in the “middle” market segments, between organic and mainstream, are characterized by national or even regional approaches. This limits the initiatives in potential scale advantages and discourages multinationals in animal-based production chains and large exporting countries to actively develop animal-friendly products. A wider presence and more European orientation of animal-interest groups would increase the potential acceptance of animal-friendly products targeted at an international market. Finally, the uneven distribution of costs and benefits of animal welfare is a potential threat for market development as farmers play a vital role in the production of animal-friendly products. All in all, a more animal-friendly future that is achieved through the market is not necessarily a utopic goal. Such a future can, however, only be created with substantial policy attention from not only market stakeholders, but also civil society and governmental stakeholders in society to guide the market in the desired direction.
